# Structure of Health Information With Different Information Models: Evaluation Study With Competency Questions

**DOI:** 10.2196/46477

**Published:** 2023-07-31

**Authors:** Anna Rossander, Daniel Karlsson

**Affiliations:** 1 Department of Applied IT University of Gothenburg Gothenburg Sweden; 2 Swedish eHealth Agency Stockholm Sweden

**Keywords:** informatics, health care, information model, terminology, terminologies, interoperability, competency question, interoperable, competency, EHR, electronic health record, guideline, standard, recommendation, information system

## Abstract

**Background:**

There is a flora of health care information models but no consensus on which to use. This leads to poor information sharing and duplicate modelling work. The amount and type of differences between models has, to our knowledge, not been evaluated.

**Objective:**

This work aims to explore how information structured with various information models differ in practice. Our hypothesis is that differences between information models are overestimated. This work will also assess the usability of competency questions as a method for evaluation of information models within health care.

**Methods:**

In this study, 4 information standards, 2 standards for secondary use, and 2 electronic health record systems were included as material. Competency questions were developed for a random selection of recommendations from a clinical guideline. The information needed to answer the competency questions was modelled according to each included information model, and the results were analyzed. Differences in structure and terminology were quantified for each combination of standards.

**Results:**

In this study, 36 competency questions were developed and answered. In general, similarities between the included information models were larger than the differences. The demarcation between information model and terminology was overall similar; on average, 45% of the included structures were identical between models. Choices of terminology differed within and between models; on average, 11% was usable in interaction with each other. The information models included in this study were able to represent most information required for answering the competency questions.

**Conclusions:**

Different but same same; in practice, different information models structure much information in a similar fashion. To increase interoperability within and between systems, it is more important to move toward structuring information with any information model rather than finding or developing a perfect information model. Competency questions are a feasible way of evaluating how information models perform in practice.

## Introduction

### Background

Increased use of standards is often suggested as part of the solution to the problem of siloed and unusable information in electronic health records (EHRs) [1], but there is no consensus yet on what standards to use [2]. Instead, there is a flora of standards and the same information is structured with different standards in different settings [3-5]. There are different types of information standards. Some standards primarily aim to structure information within systems (intraoperability), whereas some are geared toward sharing information (interoperability) [6], but often, both types of standards may be used in both settings. The standards differ between and within themselves regarding the “boundary problem” [7,8], that is, the demarcation between what information is structured with the information model and what is structured with terminology or values. The standards also differ regarding if terminology is stated or not and if so which terminology. Additionally, the terminologies are sometimes standards in themselves (eg, Logical Observation Identifiers Names and Codes [LOINC] or Systematized Nomenclature of Medicine Clinical Terms [SNOMED CT]) but sometimes system-specific or information model–specific value sets.

This combination of possibilities leads to a flora of informatics components, seemingly nonreusable between settings, as noted in previous works [2,9]. However, if the information models structure information in similar ways and there is some agreement on terminologies, perhaps a way forward would be to continue using different standards. Information exchange would be facilitated but not plug and play, as the content would be similar, and the workload of structuring health care information could be shared between users of different information models. Previous work has compared system configurations in relation to a single standard and showed that different system configurations could be unified [10,11]. Works comparing different standards have shown discrepancies in coverage and lack of alignment, primarily regarding terminologies [12,13]. Our hypothesis is that the differences between information models are overestimated. This work contributes by evaluating both the amount and type of differences between models and by providing and testing a method for comparing structure and terminology choices of different standards.

### Aim

This work aims to explore if a possible solution to the challenge of sharing information and burden of modelling work within health care would be to continue using different information models. This work also aims to assess the usability of competency questions (CQs) for the evaluation of information models within health care.

### Research Questions

The objective of this study was to answer the following 2 research questions:

1. How does the content of health care information differ between information models?
2. Is the method of CQs a feasible way of comparing content in information models?

## Methods

### Choice of CQs

There are quantitative methods to evaluate information structures in use today. For example, the CAMMS (Common Assessment Method for Standards and Specifications) [14] is an established guide in Europe for assessing a wide range of aspects with primarily a quantitative outcome. The aim of this work was, however, to examine *how* a sample of clinically relevant information is structured with different information models and not *how much* of the information the models could structure. To expose how information was structured, a method with qualitative results, that is, including structure and terminology of content, was needed. CQs have been used to evaluate ontologies for a long time [15]. In brief, the ontology is tested by selecting a relevant scenario and then posing questions to the ontology to see if and how the information needed to describe the scenario is structured within the ontology. CQs yield both quantitative and qualitative data. To our knowledge, CQs have not yet been used to evaluate the combination of information model and terminology within health care.

### Development of CQs

Domain knowledge has been used as the basis for CQs previously by, for example, Cui [16]. Guidelines are an established textual source for domain knowledge within health care, and we thus chose to use recommendations in a guideline as scenario for developing the CQs. The use of information needed to follow best practice as a starting point ensured that the study examined the tested information models regarding clinically relevant information as opposed to theoretical possibilities or boundaries. Any topic within health care could have been used as a starting point for this work. Central venous lines (also called central catheters) are one of the many domains where structured documentation could support adherence to best practice and facilitate research to develop best practice. To prevent misinterpretations due to translation during the work, we chose to work with Swedish guidelines [17]. In the chosen guideline, there were 104 recommendations that covered preparation, insertion, care, and removal of central venous catheters. Examples from the guidelines are as follows:

1. The tip of the central venous line should be placed distally in the superior caval vein or the right atrium, and the location should be controlled at the time of insertion.
2. Bandages with polyurethane film should be replaced every 3-5 days during inpatient care.
3. At emergency insertion of a central venous line, the advantages of a central venous line should be weighed against the risk of hemorrhage.

**Figure 1:**
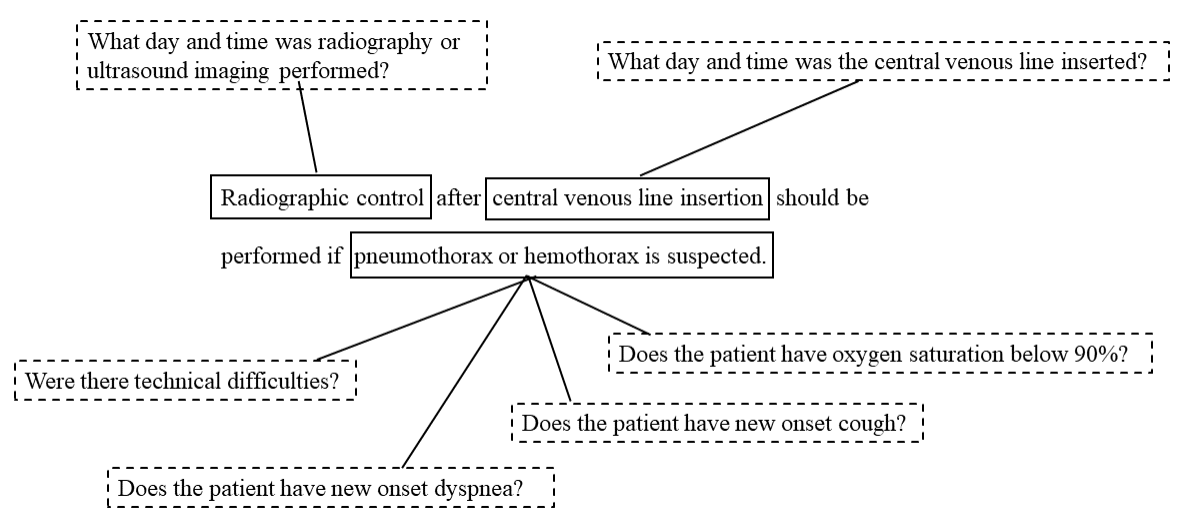
An example of a recommendation (center) with developed competency questions (in dashed boxes).

The recommendations in the guideline were graded as beneficial, equivocal, or harmful. All recommendations graded as beneficial were placed in a random order and CQs were developed iteratively from the top. Formulating the CQs is a semantic task. The recommendation was read, and if needed, divided into sections, and then questions were developed to cover all the information mentioned in the recommendation. An example is shown in Figure 1.

Recommendations that contained information not documented in the patient record, for example, “All departments using central venous lines should have access to blood- and catheter tip–culture techniques” were considered out of scope in this study and omitted. When the recommendations were not specific enough to develop CQs, the associated text in the guideline was used to interpret and operationalize the recommendation. For example, for the recommendation “Radiographic control after central venous line insertion should be performed if pneumothorax or hemothorax is suspected,” the text “Patients with pneumothorax who need treatment show new respiratory symptoms (dyspnea or cough) or oxygen saturation in blood lower than 90%” and “the risk increases with technical difficulties” was used to interpret the patients who had conditions indicative of pneumothorax or hemothorax.

The purpose of the study determines the number of CQs developed and used [18]. The focus of this work was on comparing how the different models structured clinically relevant information rather than the entire scope of each information model. During data collection, new CQs were iteratively developed and posed until further questions did not add additional types of clinical information. This is defined as the saturation point [19,20]. Despite this, a gap was discovered during data analysis regarding anatomical locations, which had not been covered by any of the initial CQs. Therefore, the next 2 recommendations in the randomized list containing information about the anatomical location were included as well. In total, 36 CQs based on 10 recommendations were developed. See Multimedia Appendix 1 for the list of included recommendations and developed CQs.

### Materials

The information models tested and compared in this study were a purposive sample. In this study, the information models are the “participants” that were selected “based on the researchers’ judgment about what potential participants will be most informative” [18]. The intention was to compare some typical models that were in use already and some models that were often recommended. The included information models differ in nature in several aspects, but they are all aimed at structuring data and thus impact interoperability. See Table 1 [21-32] for the included information models.

**Table 1:** Information models included in this study.

Information model			Description by information model provider
**Information standards**			
	FHIR^a^ [21]	FHIR is a standard for health care data exchange, published by HL7^b^ [21]	
	openEHR [22]	openEHR is a nonprofit organization that publishes technical standards for an EHR platform along with domain‑developed clinical models to define content [23]	
	HCIM^d^ [24]	HCIMs are used to capture functional semantic (nontechnical) agreements for the standardization of information used in the care process [25]	
	IPS CDA^e^ [26]	The goal of this project is to identify the required clinical data with associated vocabulary bindings and value sets for patient summary…and to build an international document and associated templates based on HL7 CDA R2…with value sets to support data elements within those templates [27]	
**Standards for secondary use**			
	OMOP^f^ [28,29]	The OMOP Common Data Model allows for the systematic analysis of disparate observational databases. The concept behind this approach is to transform data contained within those databases into a common format (data model) as well as a common representation (terminologies, vocabularies, coding schemes) and then perform systematic analyses by using a library of standard analytic routines that have been written based on the common format [30]	
	SPOR^g^ [31]	The purpose of SPOR is to, by means of integration with existing local operation planning systems, retrieve data from the perioperative process and thus offer a tool for local and national quality development (translation by authors) [32]	
**System-specific formats**			
	Electronic health record A	A health care information system used by approximately 70,000 health care staff	
	Electronic health record B	A health care information system developed and supplied by a global vendor	

^a^FHIR: Fast Healthcare Interoperability Resources.

^b^HL7: Health Level 7.

^c^HCIM: Health and Care Information Model.

^d^IPS CDA: International Patient Summary Clinical Document Architecture.

^e^OMOP: Observational Medical Outcomes Partnership.

^f^SPOR: Svenskt Perioperativt Register.

Information about the information standards and standards for secondary use were sought on publicly available sources online. Fast Healthcare Interoperability Resources (FHIR) and openEHR have national and local profiles in addition to the internationally published standards available, for example, on Simplifier [33] and in national or local clinical knowledge manager repositories [22]. An initial survey of these resources did not show profiles directly focused on the application domain, and hence, these resources were not included. The information standards are in continuous development; the latest available version was used and cited (see individual references). Draft versions were included when there was no published version of a relevant component. The Health and Care Information Models (HCIMs) are a precursor for International Organization for Standardization (ISO) 13972 [34], which were not yet published as a standard when work began, and they were therefore used as an example of that standard. The International Patient Summary (IPS) is published as both Clinical Document Architecture (CDA) and FHIR. Since FHIR was included separately, the IPS CDA format was chosen.

Templates from 2 EHR systems were included. The material consisted of locally configured user interfaces of the systems and not an information model or database model; thus, the types of results differ between the 2 EHR systems and the other included models. Further, the 2 EHR systems offer a wide possibility for users to configure templates paired with limited reference information models, and thus, the results in this work provide examples of use in the selected EHR systems. The results might have been different if an application domain other than central venous lines had been used.

SPOR (Svenskt Perioperativt Register) and the 2 EHR systems often structure the information according to the specific situation where the templates are used, as opposed to the information standards and OMOP (Observational Medical Outcomes Partnership), which are intended to be general purpose. Thus, for SPOR and the EHR systems, we have included examples of data elements where generic elements do not exist. For example, SPOR had a data element for “kind of venous access,” with access devices in the value set, where the information standards often had a generic device type data element, which could hold any device type.

### Answering the CQs

A table with the recommendations and corresponding CQs was developed. For each model, the authors together modelled the information needed to answer the CQs based on information available on the internet about the models. Both the structure of the information model, that is, what archetype/profile/entry and element was used, and terminological content, that is, what code/codesystem/unit, was documented. As an illustration, the answers for the CQ “Does the patient have new onset dyspnea?” are displayed in Table 2. For details, please see Multimedia Appendix 2. Note that the CQ does not specify what “new” means, that is, in terms of hours or days, but to determine if a symptom is new by any definition, the time of onset is needed.

**Table 2:** Example results for “Does the patient have new onset dyspnea?” for selected information models.

Element and value		Value set
**FHIR^a^ condition resource**		
	condition.code = 267036007 |Dyspnea (finding)|	SNOMED CT^b^ descendants of 404684003 |Clinical finding (finding)| (Example)
	condition.onsetTime	ISO^c^ 8601
**IPS CDA^d^ IPS problem entry**		
	hl7:value = 267036007 |Dyspnea (finding)|	SNOMED CT CORE Problem List Disorders (preferred)
	hl7:effectiveTime	ISO 21090 → ISO 8601
**OMOP^e^ condition occurrence**		
	condition.concept.ID = 267036007 |Dyspnea (finding)| (no code for dyspnea in ICDo3)	SNOMED CT or ICDo3^f^
	condition_start_date or condition_start_datetime	ISO 21090 → ISO 8601

^a^FHIR: Fast Healthcare Interoperability Resources.

^b^SNOMED CT: Systematized Nomenclature of Medicine Clinical Terms.

^c^ISO: International Organization for Standardization.

^d^IPS CDA: International Patient Summary Clinical Document Architecture.

^e^OMOP: Observational Medical Outcomes Partnership.

^f^ICDo3: International Classification of Diseases for Oncology Third Edition.

For some standards, the same information could be structured in several ways. For example, with FHIR, the information needed to answer, “What day and time was the central venous line inserted?” could be structured with both a Procedure Resource and a DeviceUseStatement. With openEHR, both Evaluation Medical Device and Action Procedure could be used. In these cases, all options were documented as results. The answers to the questions were influenced by the knowledge of the modelers answering them. The background knowledge that the authors have together was estimated to be comparable to that of a system implementer. One of the authors (DK) is, by training, a computer scientist and health informatician with experience in, for example, European Committee for Standardization and ISO standards and EHR system configuration as well as SNOMED CT. The other author (AR) is a medical doctor and health informatician with experience in structuring quality registers, SNOMED CT, and EHR system configuration. Since the authors performed the modelling, they were both researchers and participants at the same time. This gave extra insights and understanding of the work performed but also introduced a risk of bias. However, none of the authors have either any background or held any position with any of the above organizations that biased the results in any way.

### Assessment of Coverage

The models were graded for content coverage by type of clinical information. Coverage was graded into “structured” if the information needed to answer the CQs for that type of information was structured. It was graded “partially structured” when only parts of the information were structured. “Not structured” was used when there was no structure and “missing” when the information was not present in the information model.

### Assessment of Content Differences

Tables were developed for structure and coding. For each combination of models, the way or different ways the information was structured was evaluated. The number of possible ways for each model was used as the denominator and the number of ways that were similar enough to be used in interaction with the other model was used as the numerator, and the ratios of the 2 compared models were multiplied. For example, procedure type could be structured in only 1 way in FHIR but in 2 separate ways in SPOR (see Table S1 in Multimedia Appendix 2)—all 3 had a similar distribution of information between element and value, and this thus gave the following result: 1/1 × 1/2 = 50%.

Another example is procedure status where HCIM had no status field but instead used a time stamp (see Table S2 in Multimedia Appendix 2). The other models, if anything, had a coded value for status, and the result for HCIM was thus 0 for all combinations for this type of information: 0/1 × x/y = 0%.

The value sets were assessed separately in the same fashion. Where a model had several possible terminologies, those that would have been used for the information needed to answer the CQs were used. The SNOMED CT Global Patient Set [35] and full SNOMED CT were considered usable in interaction, and LOINC terms that were in the same LOINC group were also considered sufficiently similar. For model-specific value sets, common in, for example, status elements, the included values were compared and if they were equivalent, this counted as usable in interaction. All binding strengths have been assigned equal weight. When no value set was recommended, this was given the value 0.

### Definitions

**Figure 2:**
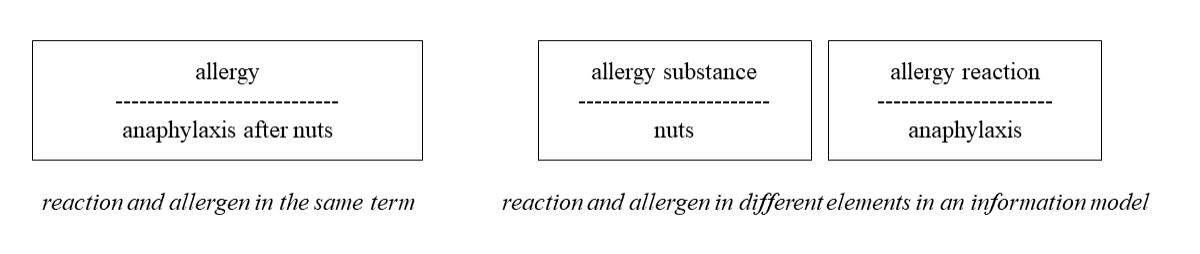
Examples of different ways of using information model and terminology in conjunction.

Information models, terminologies, and ontologies are all developed to structure information about things. In some subject areas, ontologies are in themselves sufficient to structure most information, whereas in health care, information models and terminologies or ontologies are usually used in conjunction. A division between information model and terminology is useful, as it makes the requirements on the terminology less complex. However, it leads to what is sometimes called the boundary problem, that is, the difficulty of deciding what information should be structured with the information model and what information should be structured with the terminology [7,8] (see example in Figure 2).

When not otherwise stated, we use the term information model for an information model, including its terminology bindings, that is, the terminologies or ontologies stated in it, when present. The term element is used for parts of the information model, sometimes also called attributes or headings. A value set is the stated terms or codes that are allowed for a certain element. Sometimes, a value set included entire terminology, for example, LOINC, International Classification of Diseases Tenth Revision (ICD-10), or SNOMED CT. Value set specifications may provide a binding strength to describe the flexibility with which members can be used while being compatible with the value set definition. For example, the FHIR framework provides 4 levels of binding strength: required (value set cannot be changed, eg, by extension), extensible (value set can be extended), preferred (the value set is recommended but not mandatory), and example (value set is an example only) [36]. For easier comparison of results, we have interpreted the binding strengths in the information models and described them using the FHIR definitions above.

## Results

### Research Question 1: How Does the Content of Health Care Information Differ Between Information Models?

The answers to the CQs included repeating types of information. The types of information are listed in Table 3, and the results below are presented per type. Note that the results depict the information needed to answer the CQs and not an overall evaluation of that information type.

**Table 3:** Types of information.

	Explanation	Example of competency question
Time and period	Point or period of time	What day and time was the central venous line inserted?
Procedures	Type, location, and status	Has the patient undergone radiography or ultrasound imaging?
Conditions	Type and status	Does the patient have renal impairment?
Causalities	Linking between elements or a specific causality element	Is the catheter occlusion considered due to thrombotisation?
Medications	Medicinal product	Has the patient received anticoagulant therapy?
Device types	Type or model of device used	What type of dressing was used?
Results of examinations	Type of examination and result	Does the patient have oxygen saturation below 90%?
Complex professional judgments	Assessments based on several discrete inputs and medical experience, often accompanied by motivation and degree of certainty	Is the patient’s life at risk? What is the patient’s need for central venous access?

### Coverage

**Figure 3:**
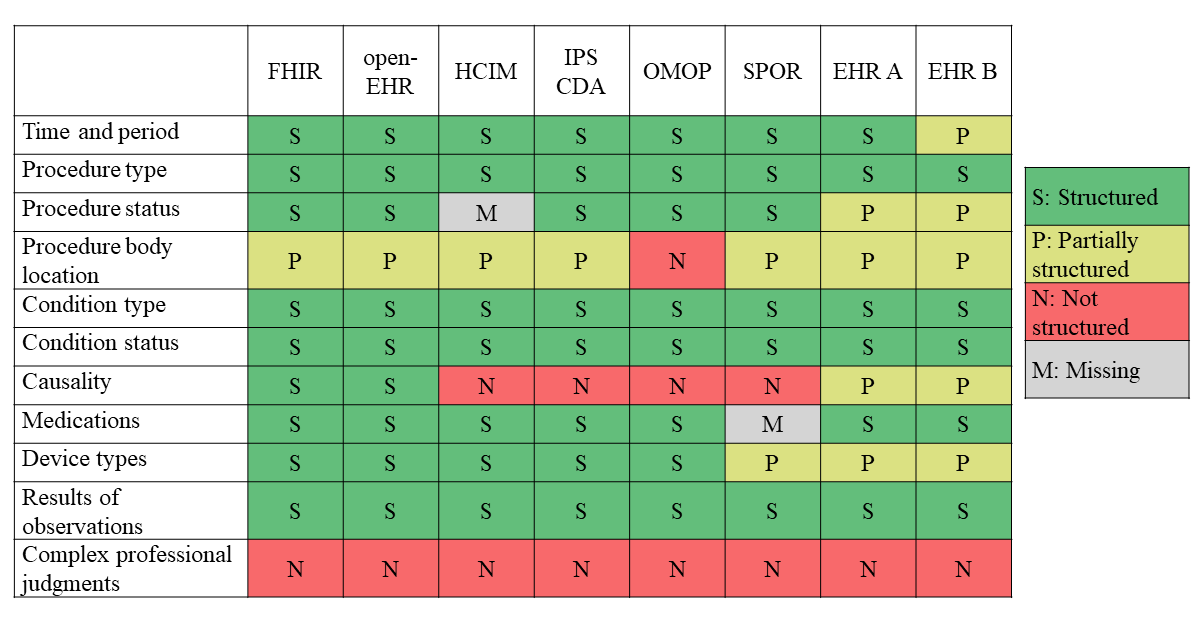
Coverage per information model for competency questions. EHR: electronic health record; FHIR: Fast Healthcare Interoperability Resources; HCIM: Health and Care Information Model; IPS CDA: International Patient Summary Clinical Document Architecture; OMOP: Observational Medical Outcomes Partnership; SPOR: Svenskt Perioperativt Register.

Results regarding coverage, that is, what information the included models had capacity to structure in a way that allowed answering of CQs, is provided per information model and type of information in Figure 3. With some exceptions, the differences in coverage were small between the included information models. The information standards and OMOP had the broadest coverage, providing structure for most types of information. SPOR could only structure information that was requested when the registry was constructed. The EHRs could hold more information than the table implies, but some of the information was locked into structures, making it difficult to find or use it in other situations. For example, in EHR B, information about radiographic control after insertion of a central venous catheter could be found under the heading “use,” where the options were “accepted for use,” “may be used before radiographic control,” “may not be used before radiographic control,” and “other.” Structures like this in EHR B were designed per instantiation and thus likely vary between settings.

### Content Differences

**Figure 4:**
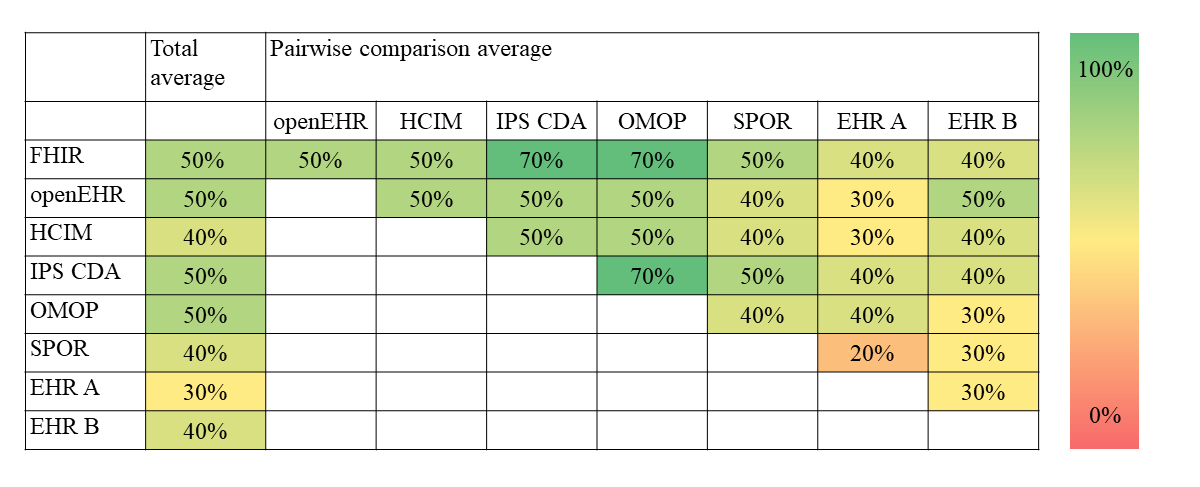
Percentage of identical structures of information. EHR: electronic health record; FHIR: Fast Healthcare Interoperability Resources; HCIM: Health and Care Information Model; IPS CDA: International Patient Summary Clinical Document Architecture; OMOP: Observational Medical Outcomes Partnership; SPOR: Svenskt Perioperativt Register.

**Figure 5:**
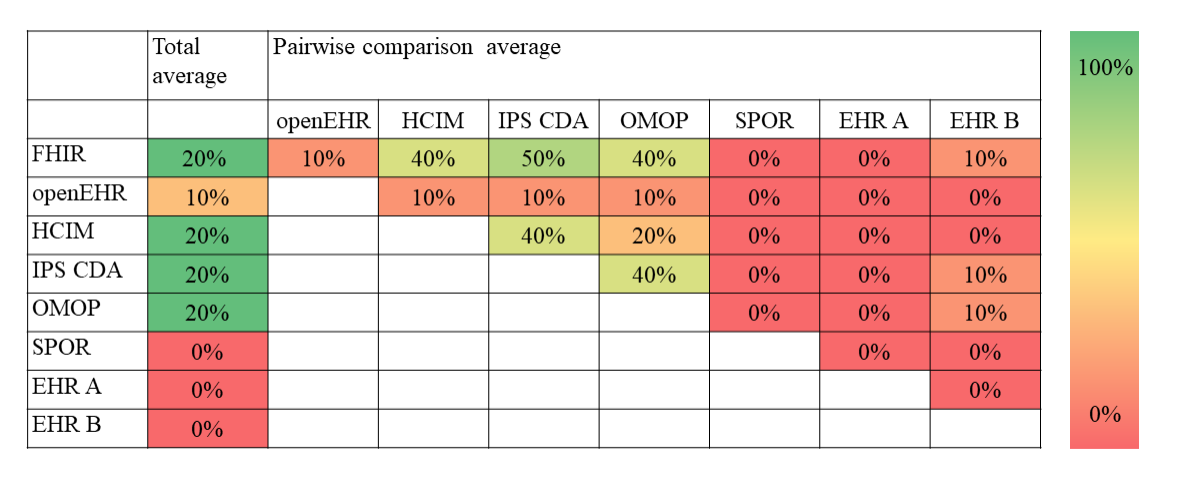
Percentage of terminologies usable in interaction with each other. EHR: electronic health record; FHIR: Fast Healthcare Interoperability Resources; HCIM: Health and Care Information Model; IPS CDA: International Patient Summary Clinical Document Architecture; OMOP: Observational Medical Outcomes Partnership; SPOR: Svenskt Perioperativt Register.

**Figure 6:**
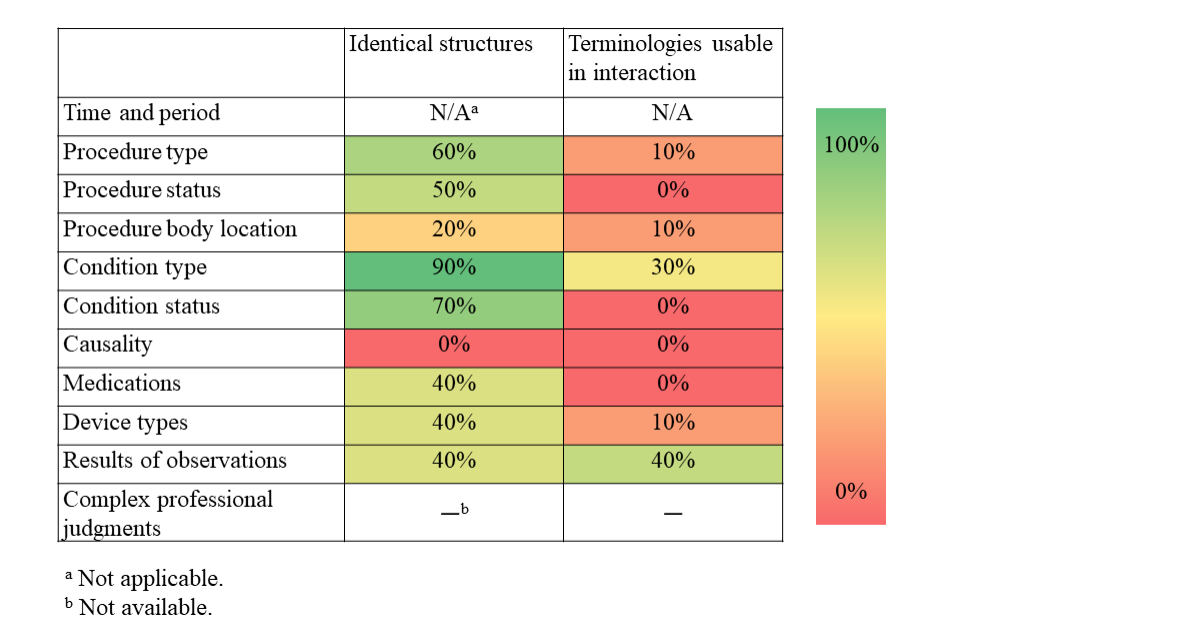
Average per information type for structure and terminologies.

Overall, the differences regarding the structure of information between the included information models were small. On average, 45% of the included structures were identical between models, that is, had the same demarcation between information model and terminology (Figure 4). The choice of terminology, however, showed a greater variation with, on average, only 11% overlap between models (Figure 5). Differences regarding structure were smaller than those regarding terminology (see Figure 6 for results per information type). Qualitative data on the content, that is, how information was structured and terminology was used, are presented as per the type of information below. The full result tables are provided in Multimedia Appendix 2. In the value set columns in the tables of Multimedia Appendix 2, all value sets listed within the information model are provided, although not all of them were relevant to the CQs. However, in our analysis, only the relevant value sets were considered.

### Time and Period

Time is repeated in many different types of structures and thus not comparable in the same way as the other information types; hence, no table for time and period is provided in Multimedia Appendix 2. The information standards and OMOP used ISO 21090 [37] and ISO 8601 [38]. Since ISO 21090 is based on ISO 8601, they are equivalent in this setting. It was not possible to determine the exact format for the Swedish SPOR or the EHRs. Some modules in EHR B only handled time of documentation, as opposed to time of the actual event, procedure, or discovered condition. There were differences between the information models on how periods were represented. FHIR and IPS CDA used interval data types, while HCIM relied on having distinct data elements for start and end points of the period. openEHR had 3 different approaches. For action archetypes, periods could be deduced from the time difference between time-stamped events. For observation archetypes, time-related information was represented through the reference model, and for evaluation archetypes, distinct data elements were used for temporal information, similar to HCIMs.

### Procedures

Information about procedures contain type of procedure (eg, insertion of central venous line), status of the procedure (eg, completed), and sometimes a location where the procedure was performed (eg, left subclavian vein). In FHIR, openEHR and OMOP procedures using a device could also be structured with the device as central information (for results regarding this, see “Device Types” below).

#### Procedure Type

All information standards and OMOP had a coded element within a dedicated procedure structure. SPOR and the 2 EHRs additionally had procedure-specific elements with a Boolean value. Of the information standards, none but HCIM strictly bound the procedure type to a terminology. HCIM had a binding strength of required and mandated the use of a code element in their ISO 21090–inspired CD datatype. The most commonly recommended terminologies in the included information models were SNOMED CT and the Swedish procedure classification KVÅ (Klassifikation av vårdåtgärder [39]; Swedish version and extension of Nordic Medico-Statistical Committee Classification of Surgical Procedures [40]) due to the Swedish context of some of the included information models. FHIR and IPS CDA used SNOMED CT, whereas HCIM and OMOP allowed several different value sets. The information models that used SNOMED CT pointed to different subsets. The Swedish registry SPOR and the 2 EHRs used KVÅ. They also, at times, used the term or code for the procedure as a question answered with a Boolean, for example, “C250 fluoroscopy during the procedure: yes/no.”

#### Procedure Status

All models that had a stated status used a separate element for this. HCIM had no explicit representation of procedure status; instead, time could be both in the past and future, indicating performed or planned procedures. It was unclear how planned but not performed procedures could be discerned from performed procedures as time passes. The standards for secondary use only represented performed procedures, and this was sufficient for answering the CQs in this work. The EHRs had multiple structures. FHIR, openEHR, and IPS CDA used coded text with native value sets, which were not always one-to-one mappable between each other.

#### Procedure Body Location

The body location of a procedure can be represented either within the value for the procedure type (see above) or in a separate element. All models except OMOP had one or many ways to separately structure body location. FHIR, openEHR, HCIM, and SPOR also had additional elements for laterality or location qualifiers. In FHIR and openEHR, these were placed in an extension and cluster, respectively. Many procedures have multiple possible locations, for example, regarding placing a central venous line–relevant location includes place of insertion (eg, left arm), the vessel in which the catheter is placed (eg, upper caval vein), and catheter tip location (eg, left atrium). None of the information models had a means to express the role of the body location in the procedure. All information standards, except openEHR, used SNOMED CT body structures. SPOR and the 2 EHRs used system-specific value sets.

### Conditions

#### Condition Type

The demarcation between information model and terminology was identical for conditions in all the investigated models, except SPOR, which had an additional structure with separate Boolean elements for key conditions. Most of the compared information models used SNOMED CT as terminology, followed by ICD-10.

#### Condition Status

All information standards, OMOP, and EHR B, structured the status of the condition in a separate element. The information standards had at least 2 elements to capture both status (eg, present, resolved, absent) and certainty of the status (eg, unconfirmed, established, suspected). The difference between status of a condition and the certainty of the condition can lead to ambiguities; for example, in the FHIR Condition Resource, it was possible to have an active (clinicalStatus) and at the same time refuted (verificationStatus) condition. Two of the openEHR code sets and the code sets in HCIM contained codes from SNOMED CT but used different concepts, and the value sets were fully disjoint. All other codes were information model–specific. Most code sets had different granularity, that is, number of codes, making one-to-one mapping between them difficult.

### Causality

Causality is a relation between entities where one is the cause of another, for example, that a deep vein thrombosis is a consequence of a central venous catheter. Of the included models, only openEHR had a separate element to document causality. openEHR also contained a LINK class, which would support this purpose, but there was no generic code set for the type of linking. In FHIR, there was a “dueTo” extension, which allowed linking conditions to their causes. The CDA standard had provisions for linking any CDA instance to any other instance, but this feature was not used for causality within the IPS CDA implementation guide. Both EHRs had structured lists for specific settings, for example, “reason for extraction” with local codes in the value set.

### Medications

Only information regarding the type of medicinal product has been evaluated in this work. Information structures of timing, dosage, dose form, and substances were not included. The structure of information on medications varied depending on stage in the process of medication, that is, for example, prescribing, dispensing, administration, or consumption. There were 2 general patterns: one where there was a single coded element for the medicinal product and one where there was a complex structure of multiple elements, such as active ingredient, dose, and dose form. The information models that had terminology bindings pointed to multiple terminologies, except FHIR that stated SNOMED CT. EHR A used the Anatomic Therapeutic Chemical classification system and the Swedish national medicinal products terminology [41]; the configuration for EHR B was not finished at the time of data collection.

### Device Types

Devices vary from short-time use artefacts as dressings to permanent implants as pacemakers. The results show how information about the type of devices was structured when the device was the central information. Several of the models had further elements for additional details, for example, batch number, size, or manufacturer. This was not included in this work. The information standards and OMOP had dedicated elements for devices with the name of the device in the value set, whereas SPOR and the EHRs used a terminology-bound element with a Boolean or a value set to further specify the device. FHIR, HCIM, IPS CDA, and OMOP all pointed to SNOMED CT as terminology. In general, it was also possible to document information about a device within the procedure where it was used as a distinct procedure type. This can be done with a term or concept where the device is included, for example, 1172566008 |Insertion of central venous catheter (procedure)|, but FHIR also permitted a separate element within the procedure class holding the device (in this example, 52124006 |Central venous catheter, device (physical object)|).

### Results of Observations

Common observations are bedside measurements, assessment scales, and laboratory results. Information regarding observations is often a combination of a question, a result value, and a unit. Sometimes these entities were structured in separate elements, and sometimes, a part of the information was structured by terminology binding the element. For example, a measurement of the oxygen saturation could be structured into “Measurement = oxygen saturation, value = 98, and unit = %” as well as “oxygen saturation in percent = 98.” There were 2 distinct approaches to representing the results of the observations. FHIR, IPS CDA, OMOP, and EHR A rely on external terminologies to express the type of observation, whereas openEHR and HCIM develop specific information models to express the type of observation where the name of the element was bound to a terminology. SPOR and EHR B had specific elements in the information model for observations but without any terminology binding.

### Complex Professional Judgments

None of the included models had a structured way to document complex judgments such as “How big problems can be expected if the central venous line is replaced?” or “Is the patient’s life at risk?”

### Research Question 2: Are CQs a Feasible Way of Comparing Content in Information Models?

#### Development of CQs

In total, 36 CQs covering 10 recommendations were developed (Table 4). For 7 of the recommendations, the information in the recommendation was enough to develop the CQs—a task performed in a few minutes. For 2 recommendations, additional information from the guidelines was needed. One recommendation required information on what substances were included in “ADP (adenosine diphosphate) receptor antagonists” and “novel oral anticoagulants,” which was not present in the guidelines.

**Table 4:** Information needed to develop the competency questions.

	Recommendations (n=10), n	Competency questions (n=36), n
Recommendation only	7	19
Recommendation and textual guideline	2	10
Additional information needed	1	7

As described in the Methods section, the initially assumed saturation point was revised during analysis of results, and additional CQs were developed for 2 recommendations.

#### Answering the CQs

The most effort in data gathering was spent on searching information about the information models and modelling. CQs covering information frequently documented in a structured way, for example, “Does the patient have renal impairment?“ were relatively straightforward to answer with all the included models. For information that is rarely structured, for example, “Was a micro punction needle used?” or “What problems can be expected if the central venous line is replaced?” much time was spent on searching information about the different models to minimize risk that a possible solution was missed. The amount of work performed in modelling the information needed for the CQs is comparable to that performed in a real-life setting modelling clinical information. Time consumption thus varied widely both depending on complexity of the area and how well the chosen information model handled the area.

#### Assessment of Coverage and Content Differences

The results from the modelling work were complex, especially when information could be structured in several ways with the same information model or when terminology binding included multiple value sets. This was demanding to capture in a spreadsheet, but evaluation of tools was beyond the scope of this work.

## Discussion

### Research Question 1: How Does the Content of Health Care Information Differ Between Information Models?

When compared pairwise, the 8 included models had, on average, 45% identical structures and 11% terminologies that were sharable. Most overlaps regarding structure were present between information standards. Content that is not identical can still be similar, and our assessment is that the similarities were larger than the differences between the compared information models in general. The information models included in this study could represent most information required for answering the CQs.

### Structure

**Figure 7:**
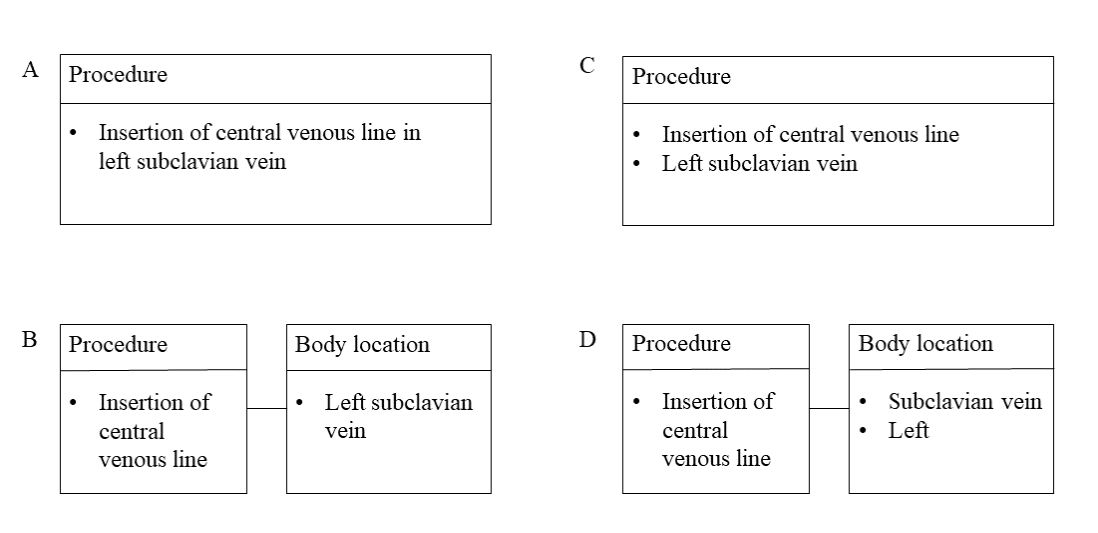
Examples of using terminology or separate elements and classes. A. Using 1 element in 1 class instance. B. Using 2 elements in 2 class instances. C. Using 2 elements in 1 class instance. D. Using 3 elements in 2 class instances.

Conditions and procedures have the highest overlap in structure. This information is thus readily sharable despite using different models if the used terminologies are the same or translatable. Representations of observations could be expected to be well-standardized due to its maturity but had only 40% overlap, mainly due to 2 different patterns of demarcation between information model and terminology. The same problem was present for medications and device types. Another common demarcation issue, present also for procedures and conditions, was that the EHRs and SPOR commonly used complex elements with a yes/no tick box, that is, with Boolean data type, whereas the information standards split the same information into several elements. Complex elements with tick boxes are tempting when developing structures for a specific use case but makes information sharing with structures developed for other use cases difficult. Conversion between these types of demarcation may be possible but includes risk of information loss and builds maintenance burden. In this material, the body locations for procedures and conditions could be represented either in a complex value for the procedure/condition itself (eg, insertion of central venous line in left subclavian vein or kidney failure) or separated in different ways (see examples in Figure 7). Similar issues can occur with other related information such as method.

When sharing information structured with different demarcations, a compositional terminology for describing both the elements of the information model and the values in the model can be beneficial [42]. A compositional terminology allows for composition and decomposition of meaning, for example, splitting “kidney failure” into “organ failure” and “kidney” or vice versa. The information standards and OMOP all refer to SNOMED CT as a possible terminology for this type of information, and SNOMED CT logic representation may, in select cases, be used to transfer between different demarcations. For this to be possible, both the element name and the value must be terminology bound to concepts or postcoordinated expressions that are logically defined in relation to each other. Some use cases such as laterality are in that sense likely to be easier to coordinate, while others may introduce significant complexities regarding postcoordination or development of new concepts. Sometimes, different elements were mandatory in different models. In such cases, it might not be possible to share data even when the information is decomposable because obligatory information might be missing.

### Terminologies

When the demarcation between information model and terminology is the same and the only difference between models is regarding terminology, information sharing possibilities depend on how easily those terminology-encoded values are converted into each other. All models used internal model-specific value sets for some elements. For example, in this material, the values for status for both procedures and conditions were different in all the included models, not only regarding terms but also by the number of values, making one-to-one mapping very difficult, not to say impossible, without information loss or distortion. Some values were, however, present in the code sets of all information models; for example, all models had a value to represent the status “the patient has this condition now” and that could thus be mapped between models. This confirms previous work, which showed that Apgar score representation had similar structures in HL7v3 DMIM (Health Level 7 version 3 Domain Message Information Model) and openEHR but were poorly bound to terminology [2] and that few value sets were aligned between the models when comparing openEHR and 3 HL7 formats for adverse sensitivity [12], although in the latter case, a joint openEHR-FHIR review has improved alignment [43].

The openEHR archetypes studied in this work were outliers compared to the other included information models in that few specific external terminologies were referred to. According to openEHR methodology, terminology binding is postponed to the templating phase, but while reviewing international templates, no additional terminology bindings were found. In this material, FHIR, HCIM, IPS CDA, and OMOP on the other hand often referred to external international terminologies, especially for larger value sets.

Where the models point to an existing terminology, there will be times when a suitable concept does not exist and therefore needs to be developed. In this material, there was, for example, no suitable concept within SNOMED CT to document the type of bandage used, despite SNOMED CT being the recommended terminology for several of the models. SNOMED CT provides the possibility to post coordinate concepts. However, postcoordination has drawbacks; for example, many health care information systems lack the capability to handle postcoordinated expressions, and postcoordinated expressions lack a human-readable term. Further, the concept model must permit the needed modelling, and the concepts needed for modelling must exist or be created [3]. Postcoordination has thus not been included as a possibility for value sets based on SNOMED CT.

Using the same terminology does not necessarily mean that the exact same code is used. For example, SNOMED CT contains 233527006 |Central venous cannula insertion (procedure)|, which has 16 more granular child concepts, and any of these could be used to document the insertion of a central venous catheter where SNOMED CT is the recommended terminology. Lack of concepts, and to an extent, the lack of capabilities to postcoordinate have led to the development of national or implementation-specific extensions to many international terminologies, including SNOMED CT and ICD-10.

### Internal Variability

The information standards aim to cover a wide range of information and offer complex structures to achieve this. They also sometimes have several different ways to structure the same information on varying levels of detail, leading to internal variability. This has been shown in evaluations of implementations of information models [13,44]. The standards for secondary use had a more rigid structure, only permitting 1 way to structure per type of information. The EHRs aim to capture all information and rely on free text to a higher degree than the other included types of models. Free text is, however, very hard to share unambiguously. Some information relevant in this work was structured very specifically in the EHRs, for example, “radiographic control before use of central venous catheter.” Other types of radiography procedures were not examined but it is unlikely that all radiography examinations are structured like this, and this is thus an example of the same procedure being structured in multiple ways also in the EHRs.

In FHIR and openEHR, structured information could be added in extensions or slots. In our material, this was found for causality (FHIR), procedure body location (FHIR and openEHR), and medication detail (openEHR). These might be tempting if the other option is free text; however, additions like this risk add to complexity and internal variability. Where information can be structured in several ways, there is a risk that instantiated information is erroneous, for example, an “upper arm fracture in the leg.” Having multiple elements to construct the meaning of a clinical statement increases the need for the sophisticated validation of information either during or after data entry to avoid mishaps.

### Areas of Poor Coverage

Complex professional judgments were not possible to structure with any of the included models. Perhaps complex professional judgments are most easily documented as free text. Placing them in a terminology-bound element in an information model would facilitate identifying and sharing the information despite it being unstructured. openEHR and FHIR were the only included models that could structure causality, both by using extension and slots, thereby opening the potential for a higher degree of variability.

### Research Question 2: Are CQs a Feasible Way of Comparing Content in Information Models?

The CQ method was a good way to probe deeper into information models from a clinically relevant perspective. The CQs revealed the types of information that were poorly structured or completely omitted—areas that are easily overseen when assessing the same information model from a theoretical perspective. One could argue that CQs leave the door ajar to bias from the evaluator, as opposed to a more formal method where the information models are described in the same format and then compared [45,46]. However, the information models relevant to compare are only available in different formalisms, often specific to the respective model, thereby restricting the use of such formal methods. Further, formal comparisons between models that differ in their demarcation between information model and terminology is not possible unless all elements are terminology bound to a machine-readable terminology, which they rarely are. Care should be taken when deciding how much effort to put into answering the CQs. Those covering information rarely structured are laborious to answer and perhaps more useful as a marker for where information is so complex that free text is the most suitable way to document it.

To cover all possible types of information, it might be necessary to push beyond the initially deemed saturation point. Perhaps, future work could use a 2-step saturation point by first developing CQs until no new types of information are uncovered and then modelling answers for the developed CQs, omitting those that duplicate already performed modelling work. This would avoid massive duplicative modelling.

### Limitations

The CQs do not push the boundaries of what the information models can handle. For example, multiple body locations or status other than “performed” or “present” for procedures or conditions are not included nor are many of the intricacies about pharmaceutical information. There might be greater differences between models than this work has revealed. The information modelling done in this work is best effort but not best possible. The modelling was not discussed with additional parties external to this study; however, this might be in correspondence with results in a real-life setting, where the amount of effort is limited by existing resources, including access to domain and informatics experts.

### Conclusions

Formal comparisons between information models show incompatibilities that are often merely theoretical [4,47], whereas practical work has shown that conversion between models for secondary use is doable [5,10,11]. This work shows that in practice, different information models structure much information in a similar fashion. To increase interoperability within and between systems, it is thus more important to move toward structuring information with any information model than finding or developing a single, perfect information model. When choosing an information model, one should consider that international standards have the best coverage and overlap between information models. They are also likely to be more widely adopted, decreasing the need for conversion before information exchange and have more users putting effort into developing them. As a final delimiter, assess the demarcation between information model and terminology and choose an information model that is similar to those of whom information is to be shared with. Put effort into decreasing internal variability and increasing terminology binding to external terminologies. The CQ method was successfully applied to the challenge of comparing health care information models. This method is a feasible way of evaluating how information models perform in practice, thereby adding valuable qualitative data on similarities and differences.

## Appendix

Multimedia Appendix 1 Recommendations and corresponding competency questions.

Multimedia Appendix 2 Structure of content per type of information.

## Abbreviations

CAMMS: Common Assessment Method for Standards and Specifications
CDA: Clinical Document Architecture
CQ: competency question
DMIM: Domain Message Information Model
EHR: electronic health record
FHIR: Fast Healthcare Interoperability Resources
HCIM: Health and Care Information Model
HL7: Health Level 7
ICD-10: International Classification of Diseases Tenth Revision
IPS: International Patient Summary
ISO: International Organization for Standardization
KVÅ: Klassifikation av vårdåtgärder
LOINC: Logical Observation Identifiers Names and Codes
OMOP: Observational Medical Outcomes Partnership
SNOMED CT: Systematized Nomenclature of Medicine Clinical Terms
SPOR: Svenskt Perioperativt Register
