# Supplementary material for: Structure of Health Information With Different Information Models: Evaluation Study With Competency Questions
Source: JMIR Med Inform. 2023 Jul 31;11:e46477. doi: 10.2196/46477 (PMC10425817; doi:10.2196/46477)
Supplement: Multimedia Appendix 1 [file medinform_v11i1e46477_app1.docx]

## Appendix 1 Recommendations and corresponding Competency Questions

Radiographic control after central venous line insertion should be performed if pneumothorax or hemothorax is suspected.

What day and time was the central venous line inserted?

Has the patient undergone x-ray or ultrasound?

What day and time was radiography or ultrasound imaging performed?

Were there technical difficulties?

Does the patient have new onset dyspnea?

Does the patient have oxygen saturation below 90%?

Does the patient have new onset cough?

At emergency insertion of a central venous line, the advantages of a central venous line should be weighed against the risk of hemorrhage.

Does the patient have warfarin treatment?

Does the patient have double trombocytic inhibitor treatment (ASA + ADP receptor inhibitor)?

Does the patient have NOACs?

What day and time was NOAC therapy withdrawn?

Does the patient have renal impairment?

What day and time was the central venous line inserted? (duplicate)

Was a micro punction needle used?

Bandage with polyurethane film should be replaced every 3-5 days during inpatient care.

What type of dressing was used?

What day was the bandage replaced?

Alteplase shall be instilled intraluminally for local thrombolysis if catheter is blocked due to thrombotisation.

Is catheter blocked due to thrombotisation?

Is the catheter occlusion considered due to thrombotisation?

Has alteplase been instilled intraluminally?

A deep venous thrombosis related to a central venous line should be treated with anticoagulant therapy.

Does the patient have venous thrombosis?

Is the deep venous thrombosis considered due to the central venous line?

Has the patient received anticoagulant therapy?

The central venous line can be removed or left in place depending on the patients need for central venous access and expected problems during a possible exchange of the venous line.

What is the patient’s need for central venous access?

What problems can be expected if the central venous line is replaced?

Thrombolysis should only be given if the patient’s life it as risk.

Is the patient’s life at risk?

Has thrombolysis been given?

Intravenous location of catheter tip should be verified clinically or radiologically at the time of insertion and before use of catheter.

Has the intravenous location of the catheter tip been verified clinically?

Has the intravenous location of the catheter tip been verified radiologically?

Has the central venous line been used?

The tip of the central venous line should be placed distally in the superior caval vein or the right atrium and the location should be controlled at the time of insertion.

Where is the tip of the central venous line placed?

Has the intravenous location of the catheter tip been verified?

When was the intravenous location of the catheter tip verified?

What day and time was the central venous line inserted? (duplicate)

Catheter exchange over guidewire may be performed if point of insertion is unaffected and there is no suspicion of catheter related infection.

Where is point of insertion?

What is status at point of insertion?

Is there a suspicion of catheter related infection?
