# Supplementary material for: Structure of Health Information With Different Information Models: Evaluation Study With Competency Questions
Source: JMIR Med Inform. 2023 Jul 31;11:e46477. doi: 10.2196/46477 (PMC10425817; doi:10.2196/46477)
Supplement: Multimedia Appendix 2 [file medinform_v11i1e46477_app2.docx]

Table S1. Content for procedure type

|  | Information model | Element | Datatype | Valueset |
| --- | --- | --- | --- | --- |
| Information standards | | | | |
|  | FHIR Procedure Resource v4.3.0[1] | code | CodeableConcept | SNOMED CT descendants of 71388002 \|Procedure (procedure)\|  Binding strength: example |
|  | openEHR  Procedure Archetype  v1.4.3 [2] | Procedure name | DV_TEXT | “Coding of the specific procedure with a terminology is preferred, where possible.” |
|  | HCIM  Procedure  v5.3[3] | ProcedureType | Inspired by CD | - CBV codes (the procedures file)  - NHG tabel 49 Ingrepen en behandelingen (procedures list of the Dutch College of General Practitioners)  - DHD Verrichtingenthesaurus (DHD procedure thesaurus)  - GGZ Verrichtingenijst (Dutch Mental Health and Addiction Care procedures list)  - SNOMED CT concepts in: Dutch obstetric procedures simple reference set  - NZa codes (Care activities file)  Binding strength: required |
|  | IPS CDA Procedure Entry[4] | code | CD.IPS | SNOMED CT GPS code set or Absent or Unknown Procedures  Binding strength: preferred |
| Standards for secondary use | | | | |
|  | OMOP PROCEDURE_OCCURRENCE Table S[5,6] | procedure_concept_id | integer | SNOMED CT, CPT4, HCPCS, ICD10PCS, ICD9Proc, OPCS4  Binding strength: required |
|  | SPOR[7] | Separate elements for key procedures | Boolean with null flavour | Elements with local codes or KVÅ Binding strength: required |
|  |  | main procedure, additional procedure | code | KVÅ Binding strength: required |
| System-specific formats | | | | |
|  | EHR A | KVÅ  procedure type can be inferred from local element names  elements with local codes; multiple choice or Boolean | | |
|  | EHR B | KVÅ  elements with local codes; multiple choice | | |

Table S2. Content for procedure status

|  | Information model | Element | Datatype | Value set |
| --- | --- | --- | --- | --- |
| Information standards | | | | |
|  | FHIR Procedure Resource[1] | status | code | preparation, in-progress, not-done, on-hold, stopped, completed, entered-in-error, unknown  Binding strength: required |
|  | openEHR  Procedure Archetype[2] | Current Action State | DV_CODED_TEXT | planned, postponed, cancelled, scheduled, suspended, aborted, active or completed  Binding strength: required |
|  | HCIM  Procedure-v5.3[3] | no status element, time used to determine status (past, current, performed) | | |
|  | IPS CDA  Procedure Entry [4] | statusCode | CS | completed, active, aborted, cancelled  Binding strength: required |
| Standards for secondary use | | | | |
|  | OMOP [5,6] | no status element, all documented procedures are performed | | |
|  | SPOR [7] | no status element, all documented procedures are performed | | |
| System-specific formats | | | | |
|  | EHR A | For some types of information, for example x-ray control, there are options: performed, to be performed, is not needed. Otherwise, all documented procedures are performed.  Binding strength: required (when present) | | |
|  | EHR B | Catheters are grouped into active or removed, all documented procedures are performed.  Binding strength: required (when present) | | |

Table S3. Content for procedure body location

|  | Information model | Element | Datatype | Valueset |  |
| --- | --- | --- | --- | --- | --- |
| Information standards | | | | | |
|  | FHIR Procedure Resource [1] | bodysite | CodeableConcept | SNOMED CT descendants of 442083009 \|Anatomical or acquired body structure (body structure)\|  Binding strength: example |  |
|  | FHIR Bodystructure Resource [8] | location | CodeableConcept | SNOMED CT descendants of 442083009 \|Anatomical or acquired body structure (body structure)\|  Binding strength: example |  |
|  |  | locationQualifier | CodeableConcept | SNOMED CT: Unilateral left, Unilateral right, Bilateral, Upper, Lower, Medial, Lateral, Superior, Inferior, Posterior, Below, Above  Binding strength: example |  |
|  | openEHR  Procedure Archetype [2] | Body site | DV_TEXT | None stated. |  |
|  | openEHR Anatomical Location Cluster[9] | Aspect | Choice of  DV_CODED_TEXT,  DV_TEXT | Medial, Lateral, Superior, Inferior, Anterior, Posterior, Proximal, Distal, Deep, Superficial, Palmar, Plantar, Dorsal, Mid, Oral, Anal  Binding strength: required (for DV_CODED_TEXT) |  |
|  | HCIM  Anatomical Location [10] | Location | CD | - SNOMED CT descendants of 442083009 \|Anatomical or acquired body structure (body structure)\|  - ICD-O-3 topography codes Binding strength: required |  |
|  |  | Laterality | CD | SNOMED CT: Left, Right, Right and left  Binding strength: required |  |
|  | IPS CDA[4] Procedure Entry | targetsite | CD.IPS | SNOMED CT descendants of 442083009 \|Anatomical or acquired body structure (body structure)\| Binding strength: preferred |  |
| Standards for secondary use | | | | | |
|  | OMOP [5,6] | No location element. | | | |
|  | SPOR[7] | Separate elements for key locations | Coded value | System-specific value set  Binding strength: required |  |
|  |  | laterality | code | Right, left, bilateral, not relevant  Binding strength: required |  |
| System-specific formats | | | | | |
|  | EHR A | Separate elements with codes from a system-specific value set | Boolean | n/a |  |
|  | EHR B | Element for Body Location | Coded value | System-specific value set  Binding strength: required |  |

Table S4. Content for condition type

|  | Information model | Element | Datatype | Valueset |
| --- | --- | --- | --- | --- |
| Information standards | | | | |
|  | FHIR  Condition Resource[11] | code | codeableConcept | SNOMED CT descendants of 404684003 \|Clinical finding (finding)\| and  160245001 \|No current problems or disability (situation)\| Binding strength: example |
|  | openEHR  Problem/Diagnosis Archetype [12] | Problem/Diagnosis name | DV_TEXT | “Coding of the name of the problem or diagnosis with a terminology is preferred, where possible.” |
|  | HCIM  Problem-v4.6[13] | ProblemName | CD | Diagnosethesaurus DHD (SNOMED CT), ICD-10, Nationale Kernset Patiëntproblemen V&VN (SNOMED CT), NANDA-I, Omaha Systems, ICF, ICPC-1 NL, G-Standaard Contra Indicaties, DSM-IV, DSM-V  Binding strength: required |
|  | IPS CDA  Problem Entry[14] | value | CD.IPS | NLM core Problem List (SNOMED CT), No information about current problems, No known problems  Binding strength: preferred |
| Standards for secondary use | | | | |
|  | OMOP  Condition Occurrence[5,6] | condition_concept_id | integer | SNOMED CT, ICDo3  Binding strength: required |
|  | SPOR[7] | main diagnose, additional diagnose | code | ICD 10,  Binding strength: required |
|  |  | indication | code | Local codes  Binding strength: required |
| System-specific formats | | | | |
|  | EHR A | ICD 10, local codes Binding strength: required | | |
|  | EHR B | SNOMED CT descendants of 404684003 \|Clinical finding (finding)\|  Binding strength: required | | |

Table S5. Content for condition status

|  | Information model | Element | Datatype | Valueset |
| --- | --- | --- | --- | --- |
| Information standards | | | | |
|  | FHIR  Condition Resource[11] | clinicalStatus | CodeableConcept | active, recurrence, relapse, inactive, remission, resolved  Binding strength: required |
|  |  | verificationStatus | CodeableConcept | unconfirmed, provisional, differential, confirmed, refuted, entered-in-error  Binding strength: required |
|  | openEHR  Problem/Diagnosis Archetype [12] | Diagnostic certainty | DV_CODED_TEXT, DV_TEXT | Suspected, Probable, Confirmed.  Binding strength: required  (for DV_CODED_TEXT) |
|  | openEHR Problem/Diagnosis qualifier Archetype[15] | Diagnostic status | DV_CODED_TEXT | SNOMED CT: Preliminary, Working, Established, Refuted  Binding strength: required |
|  |  | Current/Past? | DV_CODED_TEXT | SNOMED CT: Current, Past  Binding strength: required |
|  |  | Active/Inactive? | DV_CODED_TEXT | Active, Inactive  Binding strength: required |
|  |  | Resolution phase | DV_CODED_TEXT | Not resolving, Resolving, Resolved, Indeterminate, Relapsed  Binding strength: required |
|  |  | Remission status | DV_CODED_TEXT | In remission, Not in remission, Indeterminate  Binding strength: required |
|  | HCIM  Problem-v4.6[13] | ProblemStatusCD | CD | SNOMED CT: active, inactive  Binding strength: required |
|  |  | VerificationStatus | CD | SNOMED CT: Suspected, known possible, confirmed present, known absent, unknown  Binding strength: required |
|  | IPS Certainty Observation[16] | value | CD.IPS | unconfirmed, confirmed, refuted  Binding strength: required |
|  | IPS Problem Status Observation[17] | value | CE.IPS | active, inactive  Binding strength: required |
| Standards for secondary use | | | | |
|  | OMOP  Condition Occurrence[5,6] | condition_status_concept_id | code | Admission diagnosis, Cause of death, Condition to be diagnosed by procedure, Confirmed diagnosis, Contributory cause of death, Death diagnosis, Discharge diagnosis, Immediate cause of death, Postoperative diagnosis, Preliminary diagnosis, Preoperative diagnosis, Primary admission diagnosis, Primary diagnosis, Primary discharge diagnosis, Primary referral diagnosis  Binding strength: required |
|  | SPOR[7] | No status element, all documented conditions are present. | | |
| System-specific formats | | | | |
|  | EHR A | No status element, all documented conditions are present. | | |
|  | EHR B | Problems can be tagged as “this visit”, “chronic” and “historical”. Binding strength: required | | |

Table S6. Content for causality

|  | Information model | Element | Datatype | Valueset |
| --- | --- | --- | --- | --- |
| Information standards | | | | |
|  | FHIR dueTo Extension[18] | dueTo | CodeableConcept or Reference | All SNOMED CT codes  Binding strength: example |
|  | openEHR Problem/Diagnosis[12] | Cause | DV_TEXT | “Coding with an external terminology is preferred, where possible.” |
|  | openEHR link class[19] | meaning | DV_TEXT | Reference to categories from Annex C, ENV 13606-2 Binding strength: example |
|  | HCIM | No structured way to document this found. | | |
|  | IPS CDA | No structured way to document this found. | | |
| Standards for secondary use | | | | |
|  | OMOP | No structured way to document this found. | | |
|  | SPOR[7] | No structured way to document this found. | | |
| System-specific formats | | | | |
|  | EHR A | For some types of information, for example reason for extraction, there are set options: “central line not needed, central line not working, suspected infection, verified infection, change to other central venous line, other”  Binding strength: required (when present) | | |
|  | EHR B | For some types of information, for example reason for extraction, there are set options: “central line not needed, central line not working, unintentional removal, complication, other”  Binding strength: required (when present) | | |

Table S7. Content for medications

|  | Information model | Element | Datatype | Valueset |
| --- | --- | --- | --- | --- |
| Information standards | | | | |
|  | FHIR MedicationStatement Resource[20], FHIR MedicationRequest Resource[21], FHIR MedicationDispense Resource[22], FHIR MedicationAdministration Resource[23] | medicationCodeableConcept | codeableConcept | SNOMED CT descendants of 763158003 \|Medicinal product (product)\| Binding strength: example |
|  |  | medicationReference | Reference(Medication) | FHIR Medication Resource (see separate row) |
|  | FHIR Medication Resource[24] | code | CodeableConcept | SNOMED CT descendants of 763158003 \|Medicinal product (product)\| Binding strength: example |
|  | openEHR Medication Summary[25] | Medication name | DV_TEXT | “Coding of 'Medication name' with a terminology capable of triggering decision support is strongly recommended where possible, using for example RxNorm, DM+D, Australian Medicines Terminology or FEST” |
|  | openEHR Medication screening questionnaire[26] | Medication name | DV_TEXT | “Coding of the 'Medication name' with a terminology is preferred, where possible.” |
|  | openEHR Medication Statement[27] | Medication item name | DV_TEXT | “It is strongly recommended that the 'Medication item' be coded with a terminology capable of triggering decision support, where possible. “ |
|  | openEHR Medication Order[28], openEHR Medication Management[29] | Medication item | DV_TEXT | “Coding with terminology strongly recommended” |
|  |  | Medication details | Slot | openEHR  Medication details (see separate row) and specialisations |
|  | openEHR  Medication details[30] | Name | DV_TEXT | “This item should be coded if possible, using for example, RxNorm, DM+D, Australian Medicines Terminology or FEST.” |
|  | HCIM  PharmaceuticalProduct-v2.2 [31] | Medication Code | CD | GTIN International Article Number, ZI-nummer (2.16.840.1.113883.2.4.4.8), Trade product code (HPK)  Prescription code (PRK), Generic product code (GPK), Anatomic Therapeutic Classification code (ATC), Substance Name Code (SNK), Substance Name Code in combination with Route of Administration (SSK) SNOMED CT code |
|  | IPS CDA Manufactured Material [32] | .code | CE.IPS | - |
|  |  | .pharm:asSpecializedKind | CD.IPS | ATC Binding strength: required |
|  |  | .pharm:ingredient.pharm:ingridientSubstance.pharm:code | CD.IPS | SCT < 410942007 \|Drug or medicament (substance)\|  Binding strength: preferred |
| Standards for secondary use | | | | |
|  | OMOP Drug Exposure[6,33] | drug_concept_id | integer | RxNorm, RxNorm Extension, CVX  HCPCS, CPT4, HemOnc, NAAACCR, ATC  Binding strength: required |
|  | SPOR[7] | Medications are not documented | | |
| System-specific formats | | | | |
|  | EHR A Medication Component | |  | ATC and NPLid  Binding strength: required |
|  | EHR B Medication Component | |  | unknown |

Table S8. Content for device types

|  | Information model | Element | Datatype | Valueset |
| --- | --- | --- | --- | --- |
| Information standards | | | | |
|  | FHIR Procedure Resource[1] | focalDevice.manipulated | Reference(Device) | n/a |
|  | FHIR Device Resource [34] | type | codeableConcept | SNOMED CT descendants of 49062001 \|Device (physical object)\|  Binding strength: example |
|  | openEHR Medical Device Summary[35] | Device type | DV_TEXT | n/a |
|  |  | Device name | DV_TEXT | n/a |
|  | openEHR Medical Device [36] | Device name | DV_TEXT | “Coding with a terminology is desirable, where possible, although this may be local and depending on local supplies available.” |
|  |  | Type | DV_TEXT | “Coding with a terminology is desirable, where possible.” |
|  | HCIM Medical Device[37] | ProductID | ST | HIBCPrpductIDCodelist - Health Industry Bar Code (HIBC)  ProductIDCodelist - Global Trade Item Number (GTIN)  Binding strength: required |
|  |  | ProductType | CD | SNOMED CT descendants of 260787004 \|Physical object (physical object)\|  Binding strength: required |
|  | IPS CDA Medical Device[38] | hl7:participant:participantRole:playingDevice:Role | CD.IPS | SNOMED CT descendants of 49062001 \| Device \| included in Global Patient Set (~72 concepts)  OR absent or unknown Binding strength: preferred |
| Standards for secondary use | | | | |
|  | OMOP DEVICE_EXPOSURE[6,39] | device_concept_id | integer | SNOMED CT  Binding strength: required |
|  | SPOR [7] | Type of venous access (author translation) | code | Local code system  Binding strength: required |
| System-specific formats | | | | |
|  | EHR A | Device type is documented with a local element name, and further specified with values from a local code system.  Binding strength: required | | |
|  | EHR B | Device type is documented with a local element name, and further specified with values from a local code system inspired by KVÅ.  Binding strength: required | | |

Table S9. Content for results of observations

|  | Information model | Element | Datatype | Valueset | |
| --- | --- | --- | --- | --- | --- |
| Information standards | | | | | |
|  | FHIR Resource Observation[40] | Observation.code | codeableConcept | LOINC  Binding strength: example | |
|  |  | Observation.value[x] | Choice | n/a | |
|  | FHIR Oxygen Saturation Profile[41] | Observation.code | codeableConcept | Fixed code LOINC 2708-6, additional more specific codes may be used  Binding strength: preferred | |
|  |  | Observation. valueQuantity | Quantity | n/a | |
|  | openEHR Pulse oximetry [42] | SpO₂  terminology bound to  LOINC 59408-5 \| Oxygen saturation in Arterial blood by Pulse oximetry \| and SNOMED CT  431314004 \|Peripheral oxygen saturation (observable entity)\| | Proportion | n/a | |
|  | HCIM O2-saturation [43] | SpO2Value  terminology bound to LOINC 59408-5 \| Oxygen saturation in Arterial blood by Pulse oximetry \| and SNOMED CT 431314004 \|Peripheral oxygen saturation (observable entity)\| | PQ | n/a | |
|  | IPS CDA Result Observation[44] | code | CD.IPS | LOINC subset including 2708-6 Binding strength: required | |
|  |  | value | Choice | n/a | |
| Standards for secondary use | | | | | |
|  | OMOP MEASUREMENT[45] | measurement_concept_id | integer | SNOMED CT, LOINC  Binding strength: required | |
|  |  | value_as_number | float | n/a | |
|  | SPOR | SpO2 Value | integer | n/a | |
| System-specific formats | | | | | |
|  | EHR A | term | Vendor specific codes mapped to the Rosetta terminology  Binding strength: required | | |
|  |  | Measured value | Quantity | | n/a |
|  | EHR B | SpO2 Value with unknown terminology | Quantity | | n/a |

References for Appendix 2

1. HL7FHIR. FHIR v4.3.0 Procedure Resource. Available from: https://www.hl7.org/fhir/procedure.html [accessed Jul 11, 2022]

2. openEHR. OpenEHR Procedure v1.4.3. Available from: https://ckm.openehr.org/ckm/archetypes/1013.1.204 [accessed Jul 11, 2022]

3. Nictiz. HCIM Procedure-v5.4(2022EN). Available from: https://zibs.nl/wiki/Procedure-v5.4(2022EN) [accessed Jul 11, 2022]

4. International Patient Summary - Procedure Entry 2021. Available from: https://art-decor.org/art-decor/decor-templates--hl7ips-?section=templates&id=2.16.840.1.113883.10.22.4.17&effectiveDate=2020-07-14T16:35:58&language=en-US [accessed Jul 8, 2022]

5. OMOP CDM v5.4 CONDITION_OCCURRENCE. Available from: http://ohdsi.github.io/CommonDataModel/cdm54.html#CONDITION_OCCURRENCE [accessed Jul 8, 2022]

6. The Book of OHDSI - Chapter 5 Standardized Vocabularies. Available from: https://ohdsi.github.io/TheBookOfOhdsi/StandardizedVocabularies.html#concepts [accessed Jul 11, 2022]

7. Variabellistan – SPOR. Available from: https://spor.se/spor-for-dig-som/vardgivare-tekniker/uppdatera-registret/ [accessed Jul 8, 2022]

8. HL7FHIR. FHIR v4.3.0 BodyStructure Resource. Available from: https://www.hl7.org/fhir/bodystructure.html [accessed Jul 11, 2022]

9. openEHR. OpenEHR Anatomical Location v1.2.2. Available from: https://ckm.openehr.org/ckm/archetypes/1013.1.587 [accessed Aug 11, 2022]

10. Nictiz. HCIM AnatomicalLocation-v1.0.2(2022EN). Available from: https://zibs.nl/wiki/AnatomicalLocation-v1.0.2(2022EN) [accessed Jul 11, 2022]

11. HL7FHIR. FHIR v4.3.0 Condition Resource. Available from: https://www.hl7.org/fhir/condition.html [accessed Jul 8, 2022]

12. openEHR. OpenEHR Problem/Diagnosis v1.1.1. Available from: https://ckm.openehr.org/ckm/archetypes/1013.1.169 [accessed Jul 11, 2022]

13. Nictiz. HCIM Problem-v4.6(2022EN). Available from: https://zibs.nl/wiki/Problem-v4.6(2022EN) [accessed Jul 11, 2022]

14. International Patient Summary - Problem Entry 2021. Available from: https://art-decor.org/art-decor/decor-templates--hl7ips-?section=templates&id=2.16.840.1.113883.10.22.4.8&effectiveDate=2021-08-04T08:52:52&language=en-US [accessed Jul 8, 2022]

15. openEHR. OpenEHR Problem/Diagnosis Qualifier v1.0.1. Available from: https://ckm.openehr.org/ckm/archetypes/1013.1.1451/16 [accessed Aug 12, 2022]

16. International Patient Summary - Certainty Observation (STU1). Available from: https://art-decor.org/art-decor/decor-templates--hl7ips-?id=2.16.840.1.113883.10.22.4.19&effectiveDate=dynamic [accessed Aug 12, 2022]

17. International Patient Summary - Problem Status Observation (STU1). Available from: https://art-decor.org/art-decor/decor-templates--hl7ips-?id=2.16.840.1.113883.10.22.4.20&effectiveDate=2017-03-29T00%3A00%3A00 [accessed Aug 12, 2022]

18. HL7FHIR. FHIR v4.3.0 Extension: dueTo. Available from: https://www.hl7.org/fhir/extension-condition-dueto.html [accessed Aug 29, 2022]

19. openEHR. OpenEHR link class. Available from: https://specifications.openehr.org/releases/RM/latest/common.html#_link_class [accessed Nov 7, 2022]

20. HL7FHIR. FHIR v4.3.0 MedicationStatement Resource. Available from: https://www.hl7.org/fhir/medicationstatement.html [accessed Aug 12, 2022]

21. HL7FHIR. FHIR v4.3.0 MedicationRequest Resource. Available from: https://www.hl7.org/fhir/medicationrequest.html [accessed Oct 28, 2022]

22. HL7FHIR. FHIR v4.3.0 MedicationDispense Resource. Available from: https://www.hl7.org/fhir/medicationdispense.html [accessed Oct 28, 2022]

23. HL7FHIR. FHIR v4.3.0 MedicationAdministration Resource. Available from: https://www.hl7.org/fhir/medicationadministration.html [accessed Aug 12, 2022]

24. HL7FHIR. FHIR v4.3.0 Medication Resource. Available from: https://www.hl7.org/fhir/medication.html [accessed Aug 12, 2022]

25. openEHR. OpenEHR Medication Summary v0.0.1-alpha. Available from: https://ckm.openehr.org/ckm/archetypes/1013.1.2825 [accessed Aug 29, 2022]

26. openEHR. OpenEHR Medication Screening Questionnaire v1.0.0. Available from: https://ckm.openehr.org/ckm/archetypes/1013.1.4677 [accessed Aug 29, 2022]

27. openEHR. OpenEHR Medication Statement v0.0.1-alpha. Available from: https://ckm.openehr.org/ckm/archetypes/1013.1.4949 [accessed Aug 29, 2022]

28. openEHR. OpenEHR Medication Order v3.0.2. Available from: https://ckm.openehr.org/ckm/archetypes/1013.1.5946 [accessed Aug 12, 2022]

29. openEHR. OpenEHR Medication Management 1.2.0. Available from: https://ckm.openehr.org/ckm/archetypes/1013.1.123 [accessed Aug 29, 2022]

30. openEHR. OpenEHR Medication Details v2.0.1. Available from: https://ckm.openehr.org/ckm/archetypes/1013.1.5947 [accessed Aug 29, 2022]

31. Nictiz. HCIM PharmaceuticalProduct-v2.2(2022EN). Available from: https://zibs.nl/wiki/PharmaceuticalProduct-v2.2(2022EN) [accessed Jul 11, 2022]

32. International Patient Summary - Manufactured Material 2021. Available from: https://art-decor.org/art-decor/decor-templates--hl7ips-?section=templates&id=2.16.840.1.113883.10.22.4.3&effectiveDate=2021-08-02T16:52:27&language=en-US [accessed Jul 11, 2022]

33. OMOP CDM v5.4 DRUG_EXPOSURE. Available from: http://ohdsi.github.io/CommonDataModel/cdm54.html#DRUG_EXPOSURE [accessed Jul 11, 2022]

34. HL7FHIR. FHIR v4.3.0 Device Resource. Available from: http://hl7.org/fhir/device.html [accessed Jul 25, 2022]

35. openEHR. OpenEHR Medical Device Summary v0.0.1-alpha. Available from: https://ckm.openehr.org/ckm/archetypes/1013.1.2381 [accessed Jul 25, 2022]

36. openEHR. OpenEHR Medical Device 1.1.2. Available from: https://ckm.openehr.org/ckm/archetypes/1013.1.17 [accessed Oct 24, 2022]

37. Nictiz. HCIM MedicalDevice-v3.5(2022EN). Available from: https://zibs.nl/wiki/MedicalDevice-v3.5(2022EN) [accessed Jul 25, 2022]

38. International Patient Summary - Medical Device (STU1). Available from: https://art-decor.org/art-decor/decor-templates--hl7ips-?section=templates&id=2.16.840.1.113883.10.22.4.26&effectiveDate=2017-04-11T00:00:00&language=en-US [accessed Jul 25, 2022]

39. OMOP CDM v5.4 DEVICE_EXPOSURE. Available from: http://ohdsi.github.io/CommonDataModel/cdm54.html#DEVICE_EXPOSURE [accessed Jul 25, 2022]

40. HL7FHIR. FHIR v4.3.0 Observation Resource. Available from: http://hl7.org/fhir/observation.html [accessed Oct 12, 2022]

41. HL7FHIR. FHIR v4.3.0 Observation Oxygen Saturation Profile. Available from: https://www.hl7.org/fhir/oxygensat.html [accessed Oct 24, 2022]

42. openEHR. OpenEHR Pulse Oximetry v1.1.3. Available from: https://ckm.openehr.org/ckm/archetypes/1013.1.3084 [accessed Oct 28, 2022]

43. Nictiz. HCIM O2Saturation-v4.0 (2022EN). Available from: https://zibs.nl/wiki/O2Saturation-v4.0(2022EN) [accessed Oct 28, 2022]

44. International Patient Summary - Result Observation (STU1). Available from: https://art-decor.org/art-decor/decor-templates--hl7ips-?id=2.16.840.1.113883.10.22.4.20&effectiveDate=2017-03-29T00%3A00%3A00 [accessed Oct 31, 2022]

45. OMOP CDM v5.4 MEASUREMENT. Available from: http://ohdsi.github.io/CommonDataModel/cdm54.html#MEASUREMENT [accessed Oct 31, 2022]
